# Mr. Davies's Case of Tinea Capitis

**Published:** 1803-01-01

**Authors:** H. Davies

**Affiliations:** Piccadilly


### *To the Editors of the Medical and Physical Journal.*

Gentlemen,

In your last Number, a Correspondent published a Case, (with an engraving) of an inveterate *Tinea Capitis;* which, it seems, from his account, resisted the common plan of treatment, and the complaint remained nearly stationary.

This disease is always a loathsome one, and in many instances very stubborn. How far it is to be considered as merely *local,* arising from a morbid state of the glands at the root of the hair, or connected with a scrophulous taint in the system, is not my present purpose to enquire into; I shall content myself in relating a successful treatment of it, in a case where the patient had been afflicted for a great number of years; and, if the recital of it is judged to be worthy of notice, you will have the goodness to permit it to occupy a page of your useful Miscellany.

Mrs.

Mrs. B. living near Leicester Square, about twenty-six years of age, had been labouring under this offensive malady for *nine or ten years*, previous to her application to me. She had been treated for it, at different times during that period, but unsuccessfully. Upon first inspecting the head, it exhibited, certainly, a very unpromising appearance; for it was nearly all covered with a thick crust, somewhat resembling the bark of an old tree. I ordered it to be well washed with a lather of soap and water, night and morning, and to be covered with an ointment composed of the ung. picis, with the addition of a very small proportion of hydrarg. muriat. and then to apply a piece of thin oil-skin over the whole, with a view to retain the ointment on the parts affected; and, as I did not chuse to trust altogether to an external application in a case of so inveterate a kind, I prescribed a small dose of sulph. antim. precip. and calomel night and morning, with the pulv. sarsaparillæ twice a day, occasionally interposing a cathartic once or twice a week.

In a few days the crust began gradually to fall off, leaving the surface which it covered in a state of slight inflammation from the action of the ointment, but which soon subsided by applying the ungt. sperm. cet. with a few grains of cerruss. acet. Persevering in this method for three or four weeks, the head was relieved of its *noxious incumbrance*; the hair began to grow again, and the patient had the pleasing disappointment of being cured of a troublesome disorder, which she had too much reason to fear, from its unusually long standing, and the fruitless attempts formerly made, would resist all applications, and prove finally incurable.

How far the internal treatment, joined with the external, contributed to the removal of this disease, I must leave the reader to judge. I am aware, that some practitioners are of opinion, that a *local* application is all that is necessary in the case. However, as I have nothing to do with the disputatious part, I have related the plan adopted, together with the effect produced; and if it serves any purposes of utility in the management of this troublesome malady, under similar appearances, my end will be fully answered.

*Piccadilly,*
*December* 7, 1802.

I am, &c.

H. DAVIES.

